# Genomic Analyses, Gene Expression and Antigenic Profile of the Trans-Sialidase Superfamily of *Trypanosoma cruzi* Reveal an Undetected Level of Complexity

**DOI:** 10.1371/journal.pone.0025914

**Published:** 2011-10-19

**Authors:** Leandro M. Freitas, Sara Lopes dos Santos, Gabriela F. Rodrigues-Luiz, Tiago A. O. Mendes, Thiago S. Rodrigues, Ricardo T. Gazzinelli, Santuza M. R. Teixeira, Ricardo T. Fujiwara, Daniella C. Bartholomeu

**Affiliations:** 1 Departamento de Parasitologia, Universidade Federal de Minas Gerais, Belo Horizonte, Brazil; 2 Centro Federal de Educação Tecnológica de Minas Gerais, Belo Horizonte, Brazil; 3 Departamento de Bioquímica e Imunologia, Universidade Federal de Minas Gerais, Belo Horizonte, Brazil; Federal University of São Paulo, Brazil

## Abstract

The protozoan parasite *Trypanosoma cruzi* is the etiologic agent of Chagas disease, a highly debilitating human pathology that affects millions of people in the Americas. The sequencing of this parasite's genome reveals that trans-sialidase/trans-sialidase-like (TcS), a polymorphic protein family known to be involved in several aspects of *T. cruzi* biology, is the largest *T. cruzi* gene family, encoding more than 1,400 genes. Despite the fact that four TcS groups are well characterized and only one of the groups contains active trans-sialidases, all members of the family are annotated in the *T. cruzi* genome database as trans-sialidase. After performing sequence clustering analysis with all TcS complete genes, we identified four additional groups, demonstrating that the TcS family is even more heterogeneous than previously thought. Interestingly, members of distinct TcS groups show distinctive patterns of chromosome localization. Members of the TcSgroupII, which harbor proteins involved in host cell attachment/invasion, are preferentially located in subtelomeric regions, whereas members of the largest and new TcSgroupV have internal chromosomal locations. Real-time RT-PCR confirms the expression of genes derived from new groups and shows that the pattern of expression is not similar within and between groups. We also performed B-cell epitope prediction on the family and constructed a TcS specific peptide array, which was screened with sera from *T. cruzi*-infected mice. We demonstrated that all seven groups represented in the array are antigenic. A highly reactive peptide occurs in sixty TcS proteins including members of two new groups and may contribute to the known cross-reactivity of *T. cruzi* epitopes during infection. Taken together, our results contribute to a better understanding of the real complexity of the TcS family and open new avenues for investigating novel roles of this family during *T. cruzi* infection.

## Introduction

The protozoan parasite *Trypanosoma cruzi* is the etiologic agent of Chagas disease, a debilitating illness that is a major cause of morbidity and mortality in several Latin America countries. Approximately 10 million people carry the parasite, which causes 10,000 deaths annually [1]. During its life cycle, *T. cruzi* passes through three developmental stages. In its insect vectors, the parasite multiplies as extracellular epimastigotes, and in the hindgut it differentiates into non-dividing trypomastigotes. These infective forms are excreted in the feces after a blood meal and may contaminate the puncture site or mucous membranes of a mammalian host, where they can invade a variety of cell types. Inside host cells, trypomastigotes differentiate into amastigotes, which, after a limited number of cell divisions, differentiate into trypomastigotes that are released into circulation upon host cell rupture. This form can then infect another mammalian host cell or be taken by the insect vector during the blood meal, where it differentiates as epimastigotes.

The ability of *T. cruzi* to survive in the mammalian host is in part due to the presence of a diverse surface membrane coat. In fact, a remarkable feature of the *T. cruzi* genome is the massive expansion of genes that encode polymorphic surface proteins, which include the trans-sialidase and trans-sialidase like superfamily (hereafter called TcS), MASP (mucin-associated surface protein), and TcMUC mucins [2]. The TcS is the largest *T. cruzi* gene family, which has more than 1,400 genes, half of which are apparently functional. One of the most well-studied members of the TcS superfamily is the trans-sialidase (TcTS) enzyme. *T. cruzi* is unable to synthesize sialic acids de novo [3], a sugar modification present in *T. cruzi* proteins implicated in several key aspects of the *T. cruzi*-host interaction. The sialylation of the parasite surface is possible due to the activity of a modified sialidase that, instead of hydrolyzing sialic acid, transfers alpha (2–3)-linked sialyl residues from sialoglycoconjugates and proteins from the host to the parasite cell-surface mucin proteins (TcMUC) [4]–[6]. The rapid sialylation of TcMUC proteins upon cell rupture confers a negatively charged coat that protects the extracellular trypomastigotes from being killed by human anti-alpha galactosyl antibodies [7].

The TcS gene family is highly polymorphic, and only a few members have critical residues necessary for catalytic activity [8]. So far, four groups of TcS have been described based on sequence similarity and functional properties. Group I contains active trans-sialidases, namely TCNA and SAPA (shed acute-phase antigen), and TS-epi proteins expressed in the trypomastigote and epimastigote forms, respectively. Group II comprises members of the gp85 surface glycoproteins TSA-1, SA85, gp90, gp82 and ASP-2, which have been implicated in host cell attachment and invasion. FL-160, a representative of group III, is a complementary regulatory protein that inhibits the alternative and classical complement pathways. TsTc13, whose function is unknown, is the representative of group IV and is included in the TcS superfamily because it contains the conserved VTVxNVxLYNR motif, which is shared by all known TcS members [8]–[13].

The TcS family was identified in the 1980s and, after the publication of the *T. cruzi* genome [2], no comprehensive analysis of its sequences has been performed. Here, by analyzing all the full-length predicted TcS proteins present in the *T. cruzi* genome, we identified four new groups. The TcS groups were characterized based on presence of key TcS motifs, chromosomal localization, expression profile and antigenic properties. Implications of the TcS diversity for *T. cruzi* biology are discussed.

## Materials and Methods

### Sequence diversity of the *T. cruzi* TcS family

Genome information and sequences were retrieved from TriTrypDB (http://TriTrypDB.org). Only complete TcS sequences totaling 508 sequences were analyzed. The DNA and the translated sequences were aligned using ClustalW 2.0 software with the default parameters [14]. These alignments were used to calculate the total (mean) nucleotide and protein diversity using MEGA4 [15] with three different methods: p-distance (nucleotide and protein sequences), Kimura-2-parameter (nucleotide sequences) and Poisson correction (protein sequences). The diversity error was estimated using bootstrap resampling with 1,000 replications.

### Spatial projection and hierarchical clustering

To identify the clusters formed by the TcS protein sequences and by the 3′ sequences flanking the TcS coding regions (300 nucleotides downstream to the stop codons), we calculated the pairwise distance and generated the distance matrixes. The distances between the sequences were generated using the package PHYLIP [16], [17]. To provide a visual representation of each distance matrix, we used the multidimensional scaling (MDS) plot with two dimensions (2D). The K-means method [18] was used to define ten clusters. The MDS, hierarchical clustering, statistical analyses and graphing were performed using the R software platform [19].

### TcS cluster distribution on *T. cruzi* chromosomes and protein representation

To define the chromosomal distribution of the TcS groups, we used as reference the genome assembly reported in [20], where pairs of homologous chromosomes were arbitrarily built as having the same size. The chromosomal coordinates of the TcS genes, regardless from each homologous chromosome they are derived, were retrieved from the TriTrypDB (http://TriTrypDB.org) and plotted on the chromosomes. The colors of each coding region were the same as the colors used in the MDS protein clusters. The relative positions in the chromosomes were calculated by dividing the start codon coordinate of each gene by the total length of the chromosome. The values found were used to produce a histogram and to compare the distribution of each cluster and the pseudogenes on the chromosomes.

FRIP coordinates were found using the motif xRxP as a query. Only those occurrences located before the Asp-box and/or closer to the N-terminal extremity were considered. The Asp-box was found using the motif SxDxGxTW as a query, allowing up to 1 mismatch, and the TcS signature motif was searched using the VTVxNVxLYNR sequence as a query. In all query motifs, x represents any amino acid. The motifs were searched using the software PatMatch [21]. The signal peptide and the GPI anchor additional site were predicted using the software SignalP [22] and GPI-SOM [23], respectively. Repetitive sequences were identified using the AA-repeatFinder developed by our group (http://gicab.decom.cefetmg.br/bio-web). Only repeats with more than 10 amino acids were reported. The figures depicting the TcS genome distribution and the protein sequences were constructed using Perl (Practical Extraction and Report Language) scripts and the Bio::Graphics module, part of the Bioperl toolkit (http://www.bioperl.org).

### Parasite cultures and RNA extraction

Epimastigotes of the CL Brener clone of *T. cruzi* were maintained in a logarithmic growth phase at 28°C in liver infusion tryptose (LIT) medium supplemented with 10% fetal bovine serum. Amastigote and trypomastigote forms were obtained from infected L6 cells grown in Dulbecco's Modified Eagle Medium supplemented with 5% fetal bovine serum, at 37°C and 5% CO_2_, as described [24]. Total RNA was isolated using the RNeasy kit (Qiagen).

### Real-time RT-PCR

Primers specific for each cluster were designed using Allele ID 7 (Premier Biosoft, Demo version), and the primer specificity was verified using e-PCR and the entire parasite genome as a template. The primers selected are listed in Table S1. Real-time PCR reactions were performed in an ABI 7500 sequence detection system (Applied Biosystems). Reactions in triplicate were prepared containing 1 mM forward and reverse primers, SYBR Green Supermix (Bio-Rad), and each diluted template cDNA. Standard curves were performed for each experiment for each pair of primers using serially diluted *T. cruzi* CL Brener genomic DNA and were used in the calculation of the relative quantity (Rq) values for each sample. qRT-PCRs for the constitutively expressed GAPDH gene were performed to normalize the expression of the TcS genes. Results were analyzed with an ANOVA test, and graphics were constructed in GraphPad Prism 5.0 (GraphPad Inc.).

### Epitope prediction, spot peptide array and immunoblot

The 508 complete TcS proteins were submitted for linear B-cell epitope prediction using the Bepipred algorithm [25]. Peptides with 15 amino acids and with prediction scores above 1.3 were selected. Peptides with 70% identity over 70% of the peptide length with *T. cruzi* proteins other than TcS were excluded. For synthesis, we selected those peptides with higher occurrences within a group and with higher prediction scores. The peptides synthesized are listed in Table S2. The peptides were covalently synthesized in pre-activated cellulose membranes according to the SPOT synthesis technique [26]. Membranes were blocked with 5% BSA and 4% sucrose in PBS and were incubated for one hour and 30 minutes with diluted mice sera (1∶500) in blocking solution. After washing, the membrane was incubated with secondary antibody IgG (Sigma) diluted to 1∶2000 in blocking solution and, after a second washing, revealed by *ECL Plus Western blotting* (GE Healthcare). The spots were visualized by fluorescence scanning. The membrane was submitted to the same experimental conditions using sera from uninfected mice. Densitometry measures and analysis of each peptide was performed using Image Master Platinum (GE), and the relative density (Rd) cut-off for positivity was determined as 2.0. Graphics were constructed in GraphPad Prism 5.0 (GraphPad Inc.).

### Ethics Statement

All animal procedures were approved by the animal care ethics committee of the Federal University of Minas Gerais (Protocol # 143/2009).

## Results

### Sequence clustering reveals eight groups of the trans-sialidase/trans-sialidase-like superfamily (TcS) of *T. cruzi*

**Figure 1:**
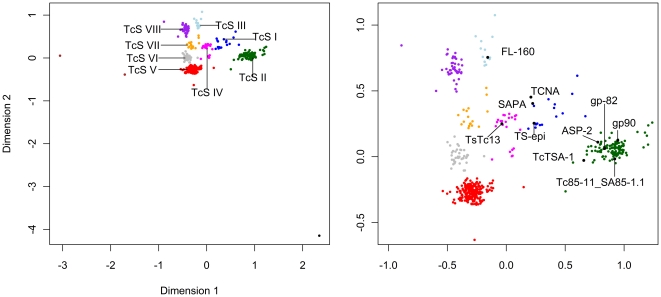
Multidimensional scaling (MDS) plot of the TcS protein sequences. The pairwise alignments of the 508 TcS complete members were performed and the distance matrix was used to generate a multidimensional scaling (MDS) plot. K-means method was used to define the clusters or groups. (A) Pattern of dispersion of all 508 TcS protein sequences resulting in 10 TcS groups. (B) Pattern of dispersion of 505 TcS protein sequences in eight TcS groups. Previously characterized TcS sequences were mapped on the MDS. TcSgroupI - blue; TcSgroupII - dark green; TcSgroupIII - light blue; TcSgroupIV - magenta; TcSgroupV - red; TcSgroupIV - gray; TcSgroupVII - orange and TcSgroupVIII - purple.

Despite the fact that four TcS groups were previously described [8], [13], [27], and only one group corresponds to the active trans-sialidase proteins, a much larger number of members of this gene family was annotated in public databases as trans-sialidases. To sort out which members correspond to the previously defined groups and to eventually identify new groups, we performed cluster analysis on all predicted TcS proteins identified in the CL Brener genome, excluding those annotated as partial and/or pseudogenes. A total of 508 TcS members were used to perform pairwise alignments resulting in a distance matrix that was used to generate a multidimensional scaling (MDS) plot (Figure 1). K-means method was then used to define ten clusters or groups (Figure 1A). Clustering with larger numbers of groups resulted in the fragmentation of previous clusters, without shuffling the members among them, indicating the robustness of the clustering of the family in ten groups (data not shown). Three members were located far from the others in the spatial distribution and therefore are the most divergent members of the family. One of them, Tc00.1047053505699.10, is the only representative of the group shown in black, and the Tc00.1047053509265.120 and Tc00.1047053507699.230 formed the brown group. Manual inspection of these three proteins revealed that their N-terminal regions are longer or shorter compared to the other TcS sequences: Tc00.1047053505699.10 contains an extra 260 amino acids at its N-terminal, whereas Tc00.1047053509265.120 and Tc00.1047053507699.230 have a deletion of approximately 160 and 450 amino acids, respectively, in their N-terminal region. The truncated sequences of these two proteins were due to the location of these genes in contig ends. Because gene prediction regarding the initial start codon could not be corrected for these three anomalous sequences, both black and brown groups were excluded from further analysis. The list of proteins belonging to each group is available in the supporting material (Table S3).

Protein and DNA sequences of the eight remaining groups were then aligned and the intra-cluster diversity was calculated using the p-distance, the Kimura-2-parameter and the Poisson correction methods, as described in the material and methods section. The groups are formed from different numbers of members and show distinct diversity indexes (Table 1). Groups labeled in red and dark green are the largest groups, with 227 and 117 members, respectively, totaling 68% of the TcS members. No clear correlation between the number of members and the diversity indexes was found. For instance, small groups (blue and orange) have similar diversity indexes of the largest ones (Table 1).

**Table 1:** Diversity indexes of nucleotide, protein and 3′UTR sequences of the TcS family.

		DNA		Protein	
	Number of members	p-distance	K2p	p-distance	Poisson correction
TcSgroupIBlue	19	0.371/0.004	0.690/0.029	0.494/0.009	0.881/0.027
TcSgroupIIDark green	117	0.264/0.004	0.340/0.007	0.419/0.010	0.558/0.018
TcSgroupIIILight blue	15	0.209/0.005	0.263/0.008	0.366/0.010	0.492/0.023
TcSgroupIVMagenta	25	0.179/0.003	0.226/0.005	0.250/0.008	0.320/0.012
TcSgroupVRed	227	0.252/0.004	0.316/0.006	0.396/0.009	0.513/0.015
TcSgroupVIGray	39	0.246/0.004	0.312/0.007	0.394/0.009	0.513/0.016
TcSgroupVIIOrange	17	0.298/0.004	0.425/0.009	0.448/0.009	0.651/0.020
TcSgroupVIIIPurple	46	0.215/0.004	0.270/0.006	0.353/0.009	0.453/0.013
TcS family	508	0.413/0.004	0.662/0.011	0.574/0.090	0.912/0.023
3′UTR	495	0.573/0.007	1.086/0.029	-	-

P-distance was used to measure the diversity of the coding regions, proteins and 3′ flanking sequences, with kimura-2-parameters and Poisson correction only to DNA coding and protein sequences, respectively.

We next mapped on the MDS plot the TcS proteins representative from each of the four previously known groups (Figure 1B). As expected, the characterized TcS members mapped into different MDS clusters. TCNA, SAPA and TS-epi, all active trans-sialidase proteins belonging to the previously defined group I, clustered together in the blue group (hereafter named TcSgroupI). From a total of 19 TcSgroupI members, 11 have the critical catalytic residues (Figure S1). GP82, GP90, Tc85-11_SA85-1.1 and ASP-2, all representatives of the previously defined group II, mapped onto the dark green cluster (TcSgroupII). Finally, FL-160 and Ts13, which belong to sialidase groups III and IV, mapped onto the light blue (TcSgroupIII) and magenta (TcSgroupIV) clusters, respectively. None of the TcS proteins previously characterized mapped onto the clusters that are red (the largest TcS group), gray, orange or purple, hereafter named TcSgroup V, VI, VII and VIII, respectively.

### Identifying key sialidase signature motifs in the eight MDS clusters

**Figure 2:**
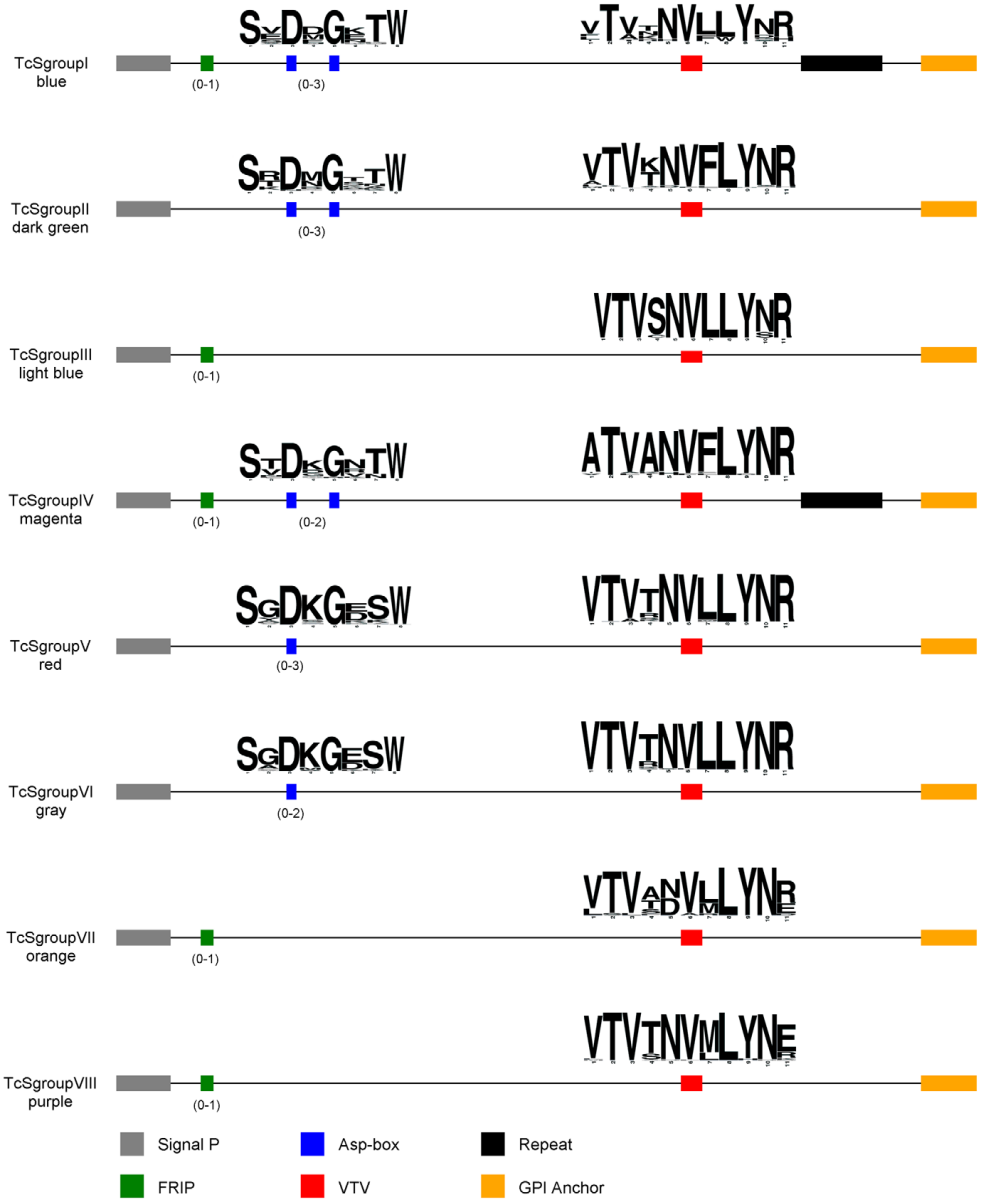
Prototype of each TcS group. The motifs are shown only when they occur in the majority of the proteins within the group. The Asp-box and VTVxNVxLYNR *logos* are shown above each motif. The numbers within parentheses indicate the number of occurrences of a given motif. The length of the proteins within the groups may vary. Graphical representations are not to scale.

To characterize each of the eight groups, we initially searched for all the key signature motifs as they are described in the literature [8], [13] and mapped them into the MDS plot (Figure S2). The canonical VTVxNVxLYNR motif was found in only 328 of 508 TcS sequences used in this study. This result prompted us to investigate whether the other proteins annotated as TcS have a degenerate form of this motif or do not have this motif at all. To this end, we performed ClustalW alignment of all 508 TcS proteins and retrieved the alignment block containing this motif. Visual inspection of this region reveals that 159 sequences have a degenerate version of this motif. Hence, 487 (96%) of the TcS sequences have the canonical or degenerate forms of the VTVxNVxLYNR motif. The remaining sequences do not contain this motif because they have a truncated C-terminal region resulting from premature stop codons and/or frameshifts. Therefore, as previously described, this motif is a signature of the TcS family that is found in all its members. As shown in Figure 2, although variations on the VTVxNVxLYNR motif are observed, the motif is highly conserved within each cluster.

We also searched for the Asp box motif found in bacterial and viral sialidases [28], using as query the SxDxGxTW sequence, where x is any amino acid. A total of 135 sequences have this motif, of which 133 belong to the previously described TcS groups I (blue), II (dark green) and IV (magenta) (Figure 2). The two other sequences having this motif belong to the TcSgroupV (red) and TcSgroupVI (gray). This result is in agreement with previous reports showing that TcSgroupIII (light blue) does not have this motif [29]. To investigate whether TcSgroupIII as well as sequences from the other four new groups have a degenerate form of the Asp box, we searched for a degenerated version of this motif, as described in the material and methods section. This search increased the number of positive Asp box sequences to 383. Only one additional degenerate position was found, resulting in the consensus SxDxGxxW. Although the majority of them have one (220) or two Asp boxes (154), a few (9) have three. Considering this new consensus motif, the Asp box is found in a large majority of the members from TcSgroupI (blue, 17 of 19 members), TcSgroupII (dark green, 114/117), and TcSgroupIV (magenta, 24/25) and is also present in the new groups TcSgroupV (red, 188/227) and TcSgroupVI (gray, 36/39). On the other hand, as previously described, it is missing in TcSgroupIII (lightblue) and has only a few occurrences in the new groups TcSgroupVII (orange, 1/17) and TcSgroupVIII (purple, 3/46) (Figure 2).

The FRIP motif was searched using the pattern xRxP (where x is any amino acid). Because this is a small and degenerate sequence, we considered only those occurrences that are before the Asp-box and/or closest to the N-terminal region [30]. A total of 205 TcS proteins contain the FRIP motif, which is found in the majority of the members of TcSgroupI (blue, 68%), TcSgroupIII (light blue, 87%), TcSgroupIV (magenta, 88%), TcSgroupVII (orange, 76%) and TcSgroupVIII (purple, 87%).

To identify repetitive regions on TcS sequences, we used the AA-repeat finder program (http://gicab.decom.cefetmg.br/bio-web). Only repeats with more than 10 amino acids were considered. We found that repeats are more frequent in the TcSgroupI (blue) and TcSgroupIV (magenta) clusters. These two groups have the largest repetitive regions, which encompass up to 884 amino acids. In fact, although we identified new repeats in these two groups, the largest repeats are those corresponding to the known DSSAH(S/G)TPSTP(A/V) repeat found in TS SAPA and the TcTs13 EPKSA-repeat. On the other hand, TcSgroupV (red), TcSgroupVI (gray) and TcSgroupVII (orange) groups have only 1, 2.5 and 6% of their members, respectively, with repetitive domains whereas no repeat was found in members of the TcSgroupIII (light blue). All repeats identified in this study are shown in Table S4.

In the prototype representation of the eight TcSgroups shown in Figure 2, it is possible to identify three patterns of motif occurrence. The TcSgroupI (blue) and TcSgroupIV (magenta) clusters have the most complex structure, with the FRIP, Asp box and VTVxNVxLYNR motifs and the C-terminal repeats, although the sequences of the VTVxNVxLYNR motif and the C-terminal tandem repeats are distinct. TcSgroupII (dark green), TcSgroupV (red) and TcSgroupVI (gray) clusters contain the Asp box and VTVxNVxLYNR motifs. TcSgroupIII (light blue), TcSgroupVII (orange) and TcSgroupVIII (purple) clusters only have the FRIP and VTVxNVxLYNR motifs, which have a consensus sequence that is group-specific. This pattern of motif occurrence is in agreement with the space distribution of the TcS groups in the MDS (Figure 1). TcS groups I and IV that have all motifs are centered in the MDS, whereas TcSgroups II, V and VI are clustered in the bottom and TcSgroups III, VII and VIII are clustered in the left top region. A graphical representation for each of the 508 TcS proteins can be found in Figure S3.

### Mapping the TcS groups on *T.cruzi* chromosomes

**Figure 3:**
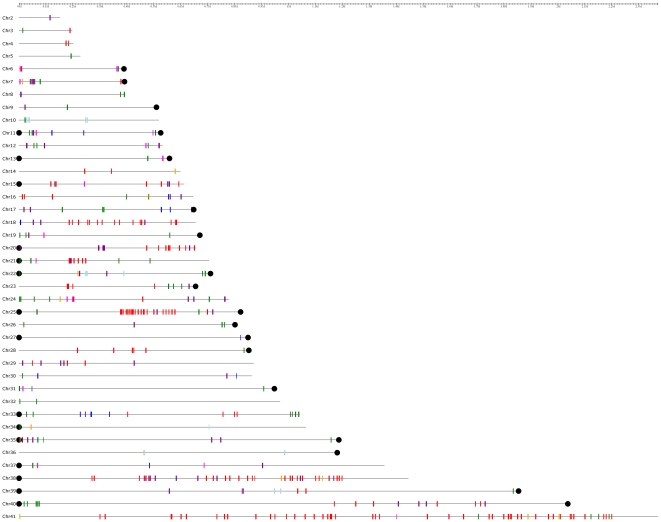
Mapping of TcS genes on *T. cruzi* chromosomes. Each CL Brener chromosome is comprised of 2 homologous chromosomes as proposed by [20]. The genes are color coded according to the color of the corresponding clusters of Figure 1. A total of 374 TcS genes could be mapped on the chromosomes. The remaining genes belong to contigs that could not be assigned to a specific chromosome, according to Weatherly et al., 2009, and are not represented in the figure. Only chromosomes containing TcS genes are shown. Black dots represent telomeric repeats. TcSgroupI - blue; TcSgroupII - dark green; TcSgroupIII - light blue; TcSgroupIV - magenta; TcSgroupV - red; TcSgroupIV - gray; TcSgroupVII - orange and TcSgroupVIII - purple.

**Figure 4:**
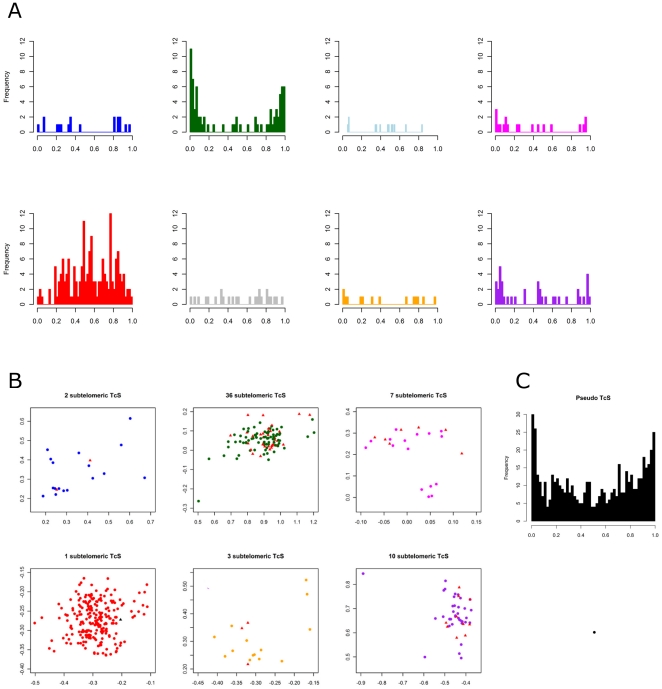
Distribution of each TcS group along the *T. cruzi* chromosomes. (A) Histograms showing the frequency of the TcS genes along the chromosomes. The length of all chromosomes was normalized as 1. The relative position of each gene was calculated by dividing the coordinate of the first nucleotide of the open reading frame by the length of the chromosome. (B) Representation of each group in Figure 1, showing the genes that localize in telomeric regions (black dots for the TcSgroupV and red dots for the other TcS groups). TcSgroupI - blue; TcSgroupII - dark green; TcSgroupIII - light blue; TcSgroupIV - magenta; TcSgroupV - red; TcSgroupIV - gray; TcSgroupVII - orange and TcSgroupVIII - purple. (C) Histogram showing the distribution of TcS pseudogenes along the *T. cruzi* chromosomes.

It is known that TcS genes can be found in *T. cruzi* subtelomeric regions or in internal positions in the chromosomes that are associated with other genes that encode surface proteins [2]. Subtelomeric regions are defined here as sequences extending from the telomeric hexamer repeats to the first nonrepetitive sequence. We investigated whether there is any bias on the chromosome localization of the TcS clusters. Figure 3 shows the chromosomal distribution of the TcS groups. A total of 60 complete TcS genes (not including partial or pseudogenes) can be found associated with the subtelomeric regions. One of them belongs to the brown cluster, which, as mentioned above, was excluded from our analysis. The majority of the subtelomeric TcS genes (36 members, 61%) belongs to TcSgroupII (dark green), 7 members from TcSgroupIV (magenta) and 10 from TcSgroupVIII (purple) (Figures 3 and 4). No TcSgroupIII (light blue) or TcSgroupVI (gray) genes are located at these regions. Interestingly, with one exception, all members of the largest TcS cluster (TcSgroup V, red) are at internal locations in the chromosomes (Figures 3, 4A and 4B). We have also found that the subtelomeric regions are enriched for TcS pseudogenes (Figure 4C), which is in agreement with the hypothesis that these regions were subject to intense rearrangement [54].

### Expression profile of the TcS genes belonging to distinct groups

**Figure 5:**
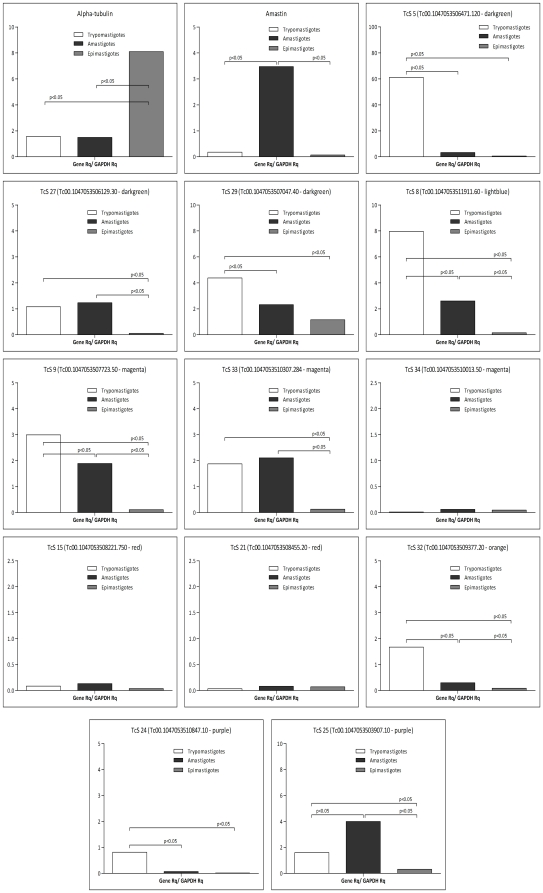
Expression profile of TcS genes by qRT-PCR. Relative quantity (Rq) calculations were based on specific standard curves for each TcS gene. Rq values of each cDNA sample (TcS Rq) were normalized with the GAPDH gene (GAPDH Rq), a gene constitutively expressed throughout the parasite life cycle. Alpha-tubulin and amastin were used as controls for genes more expressed in epimastigote and amastigote stages.

To characterize the expression profile of TcS genes belonging to distinct groups, we have performed real-time RT-PCR using member-specific primers, designed as described in the material and methods section. The expression of 12 TcS genes derived from six TcS groups was evaluated throughout the three parasite developmental stages using GAPDH mRNA levels, whose expression is constitutive throughout the parasite life cycle, for internal normalization (Figure 5). As a control, we used primers to amplify the cDNAs from the alpha-tubulin and amastin genes, whose mRNA levels we have previously shown to be up-regulated in epimastigotes and amastigotes, respectively [24], [31]. The majority of the TcS transcripts are expressed in trypomastigotes and/or amastigote forms. Interestingly, within a group, the expression profile may be highly variable. For example, the TcS5 gene that belongs to TcSgroupII is highly expressed in trypomastigote forms, whereas the TcS27 from the same group shows a much lower level of expression in the trypomastigote and amastigote forms and is barely detected in epimastigotes. Also, TcS9 and TcS33 from TcSgroupIV are more expressed in trypomastigotes and amastigotes; however, TcS34, which is from the same group, is scarcely expressed in all the development stages. The new groups also display a variable expression profile. A very low level of expression was verified for the two genes analyzed from TcSgroupV in all the developmental stages (Figure 5) as well as in the blood trypomastigotes (data not shown). On the other hand, the gene TcS32 from TcSgroupVII is more expressed in the trypomastigotes. The two members of TcSgroupVIII show a variable expression profile, with TcS24 more expressed in trypomastigotes and TcS25 more expressed in amastigotes.

### Analyzing sequence conservation of the 3′ flanking region of TcS groups

**Figure 6:**
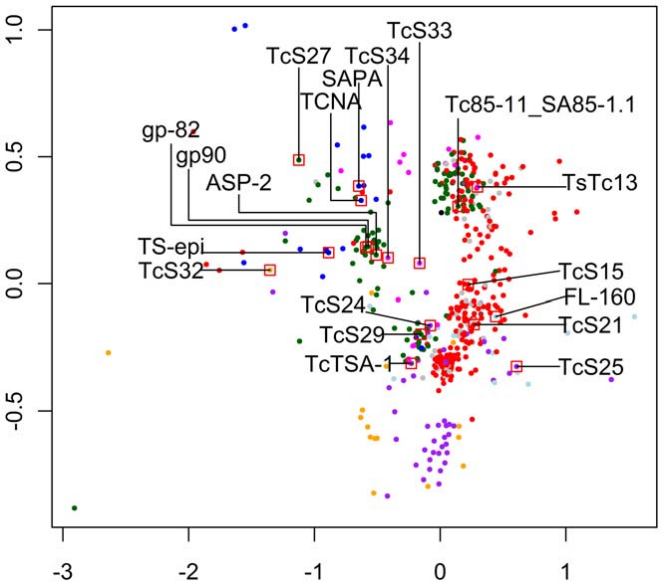
Multidimensional scaling (MDS) plot of the 3′ flanking regions of the TcS genes. A total of 300 nucleotides downstream from the stop codon of each gene were analyzed. Sequences smaller than 300 nucleotides were excluded. Previously characterized genes were mapped on the MDS.

It is well established that, in Trypanosomatids, the 3′UTR regions are involved in post-transcriptional control mechanisms that confer stage-specific gene expression. To investigate whether the 3′ flanking sequences of TcS genes that belong to the same groups are conserved, we performed pairwise alignments of the 300 nt downstream of the stop codon of the TcSs, and the distance matrix was used to generate the MDS projection. We decided to analyze 300 nt downstream from the stop codon because this is the mean average length of the *T. cruzi* 3′UTRs [32]. The sequences were then color-coded according to the protein clusters showed in Figure 1. TcS genes already characterized as well as those genes whose expression levels were analyzed by real-time RT-PCR (Figure 5) were then mapped onto the MDS projection (Figure 6). We could not find a very clear association between the protein and the 3′ flanking region distances. For example, members of the TcSgroupV (red) form a robust cluster at the protein level and are much more variable according to the analysis of the 3′ flanking region. Also, the 3′ flanking regions of TcSgroupII (dark green) members are scattered in three MDS areas. On the other hand, the 3′ flanking regions of the TcSgroupVIII (purple) members clustered together, which suggests that similar mechanisms may control the expression of some of their genes. Interestingly, the 3′ flanking regions of SAPA and TCNA, both active trans-sialidase enzymes expressed in the trypomastigote forms (TcSgroupI), are clustered very close. Also, the 3′ flanking region of the TS-epi, an active trans-sialidase that is expressed in the epimastigote stage that also belongs to TcSgroupI, is located farther away from the SAPA and TCNA sequences. Moreover, the 3′ flanking region of gp90 and gp82, both expressed in the metacyclic trypomastigotes, and ASP-2, expressed in the amastigote stage, all belong to TcSgroupII and are very close in the MDS projection. Interestingly, although Tc85-11_SA85-1.1 and TsTc13 are expressed in the trypomastigote stage, they are divergent at the protein level (Figure 1) and belong to different TcS groups (II and IV, respectively); they have similar 3′ flanking regions, which suggests that similar mechanisms for gene regulation may act on both genes.

### Antigenicity of the TcS groups

**Figure 7:**
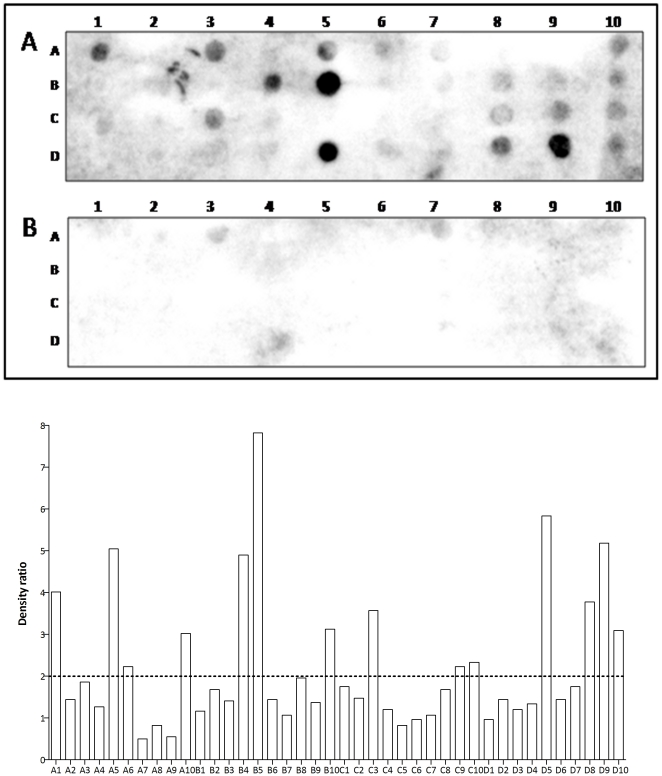
Antigenic profile of TcS peptides. The top panel shows a representative result of immunoblot employing a SPOT synthesis membrane and pools of sera from *T. cruzi*-infected mice (A) and from control uninfected mice (B). The reaction was revealed with secondary anti-total IgG antibody. The bottom panel shows the relative intensity of the signal of each spot estimated based on a comparison of the reactivity in immunoblots with sera from *T. cruzi*-infected mice to the background levels, determined by reactivity with sera from uninfected mice. A signal was scored as reactive when relative intensity (RI)≥2. The peptides analyzed for each TcS group are as follows: TcS group I, D5–D10; TcS group III, C9-D4; TcS group IV, B5-C1, C3; TcS group V, A1, C2, C3, C7; TcS group VI, C2–C8; TcS group VII, A9-B4; TcS group VIII, A2–A8.

Because the antigenicity of some members of the sialidase family was already reported [33], [34], we decided to investigate whether other peptides derived from the TcS family are also antigenic. To this end, we have performed linear B-cell epitope prediction on all 508 complete members of the TcS family. A total of 40 peptides with 15 residues, high prediction scores and high occurrences within the TcS group were synthesized in a solid support by the spot synthesis technique and screened with sera from animals infected with *T. cruzi*. The list of all peptides used in this study is shown in the Table S2. As shown in Figure 7, 11 TcS peptides derived from distinct groups displayed antigenic properties based on a cut-off signal well above background. In agreement with previous studies, peptides corresponding to the SAPA (D5 and D8) [33] and to the TsTc13 repeats (B5) [34] are highly antigenic. We have also identified new epitopes specific to the previously characterized TcSgroups I and IV (D9 and D10, and B10, respectively). At least one peptide from each of the new TcSgroups -V, VI, VII and VIII - was recognized by sera of infected animals (A1, C3, A10 and B4, and A5, respectively). The peptide C3 occurs in the largest number of members (60 in total) from the new TcS groups V and VI and from the previously characterized TcS groups II and IV.

## Discussion

The TcS superfamily, the largest *T. cruzi* multigene family [2], was described more than 20 years ago and, after the *T. cruzi* genome release, no comprehensive analysis of the diversity of this gene family was reported. Here, by analyzing all the 508 TcS complete genes present in the *T. cruzi* CL Brener genome [2], we demonstrated that this family displays an even greater variability than previously thought, as shown by means of the diversity indexes and the MDS projection. Based on their pattern of dispersion, we identified eight groups of TcS sequences, four of which were never described before (Figure 1). The distances among the clusters are consistent with the level of similarity and function of the previously described TcS sequences. All proteins that display trans-sialidase activity clustered together (TcSgroupI, blue). Another cluster was formed (TcSgroupII, dark green) from TcS proteins that have no trans-sialidase activity but that are capable of binding to β-galactose, laminin [35], fibronectin [36], collagen [37], [38], and cytokeratin [39] and are involved in cell adhesion and invasion. The third TcS group encompasses proteins involved in the regulation of the complement system (CRP - complement regulatory proteins). Previously characterized members of this group are the CRPs [29], [40], which include the FL-160 [41]. Recently, using data from the *T. cruzi* CL Brener genome project, Beucher and Norris (2008) identified CRP paralogs based on sequence similarity with a functional characterized CRP (GenBank accession number AAB49414). Also, these authors divided the CRPs into two groups, HSG (high similarity group, with more than 80% identity with AAB49414) and LSG (low-similarity group, with sequence identity between 54 and 62% with AAB49414) [29]. Here we could verify that all HSGs, and excluding two exceptions, the LSGs, fell into TcSgroupIII (light blue). These two members, which do not belong to TcSgroupIII, were clustered within TcSgroupVII (orange). In fact, they are the two most divergent sequences of the LSG subgroup [29] and correspond to members of the TcSgroupVII that are closest to the TcSgroupIII (Figure S4). Further investigation is necessary to verify whether these two proteins as well as other members of the TcSgroupVII have complement regulatory activity. Finally, a member of the TcSgroupIV that was previously described corresponds to the TsTc13 family, whose function is unknown. Based on the pattern of dispersion of the TcS groups in the MDS projection and the occurrence and sequence of key TcS motifs, we hypothesize that the new groups V and VI and the previously described TcS groupII are more related among each other when compared to the other groups. The same is valid for the new groups VII and VIII and the TcS group III. For instance, TcS groups II, V and VI are the only ones that do not have the FRIP motif and their consensus sequences of the VTVxNVxLYNR motif are very similar. Also, TcS groups III, VII and VIII share the same pattern of motif occurrence and are clustered in a similar region in the MDS projection.

*Trypanosoma brucei* genome encodes active trans-sialidases expressed in the insect form of the parasite [42]. Although no active trans-sialidase was identified in *Trypanosoma rangeli*, sialidases/sialidase-like proteins similar to TcS groups I, II and III were found, and several of these members are expressed in the epimastigote and trypomastigote forms of the parasite [43], [44]. The evolution of the TcS family suggests a gene ancestor encoding an active trans-sialidase expressed in insect forms of the genus *Trypanosoma* and several rounds of duplication and diversification would give rise to trans-sialidases expressed in mammalian forms [45]. Later in evolution *T. rangeli*, would have lost the active trans-sialidase, retaining the sialidase activity. These evidences along with the centered location of TcSgroupI in the MDS projection suggest that extensive expansion and sequence diversification of trans-sialidases similar to TcSgroupI would have originated other groups and functions.

Although the TcS family displays a high degree of sequence variation (Table 1), several motifs are conserved. The most conserved is the VTVxNVxLYNR motif, which is located upstream from the carboxyl terminus of all the TcS full-length members (Figure 2). Recently, it has been demonstrated that a version of this motif (VTVTNVFLYNRPLN), referred to as the FLY motif, may act as a virulence factor [46], [47]. BALB/c mice administered with FLY-synthetic peptide are more susceptible to *T. cruzi* infection, displaying increased systemic parasitaemia and mortality [47]. Also, it has been shown that the FLY motif binds to endothelial cells of the heart, suggesting that it might contribute to the parasite tropism to this organ [10]. We identified the exact sequence of the FLY peptide in 28 members of TcSgroupII. Because a very similar version of this motif (**A**TV**A**NVFLYNRPLN, in which mismatches are indicated in bold and are underlined) is also found in 23 members of TcSgroupIV, we speculate that, as several TcSgroupII members, this group may also participate in host cell attachment/invasion.

Two other motifs, FRIP (xRxP) and Asp box, can be found in various groups of the TcS family. The FRIP motif, which is closest to the N-terminal, is involved in binding the carboxylate group of sialic acid [48]. This motif is found not only in TcSgroupI, but also in the majority of the members of the TcS groups III, IV, VII and VIII (Figure 2). Although this motif is involved in binding sialic acid, it has been shown that enzymatically inactive members of the sialidase family in *T. cruzi* still preserve carbohydrate binding properties [49], [50]. The Asp box follows the FRIP motif and can be repeated up to five times in the sequences of viral, bacterial, trypanosomatid and mammalian sialidases. Although its function is unknown, it is worth noting that the Asp box occurs in secreted proteins and in proteins that act on, or interact with, carbohydrates [51]. Recently, it has been shown that at least some inactive trans-sialidases act as lectin-like proteins able to interact with the carbohydrate portion of glycoconjugates, only if they are sialylated [52]. The authors hypothesized that these inactive trans-sialidase proteins could bind to host surfaces that are rich in sialyl-donor glycoconjugates (functioning as anchors), facilitating the active enzyme to more efficiently undertake the sialyl-transferring activity. Here, we have shown that, in addition to TcSgroupI, members of TcSgroupIV have both FRIP and Asp box motifs (Figure 2) and therefore may also display carbohydrate binding properties.

After mapping all TcS groups on the *T. cruzi* chromosomes [20], we found no association between a group and a specific chromosomal location (Figure 3). Interestingly, we found a distinctive pattern of gene distribution along the chromosomes for members of the TcS groups II and V, with the former clearly enriched at the end of the chromosomes, whereas the latter is concentrated in the middle of the chromosomes (Figure 4). *Trypanosoma brucei* and *Plasmodium falciparum* have a sophisticated strategy for immune evasion, known as antigenic variation, which allows the parasites to adapt to the host environment through exposing and changing specific variable antigenic surface proteins [53]–[58]. In these parasites, the genes that encode surface proteins that are involved in antigenic variation are preferentially located at subtelomeric regions because these are favorable genomic environments that facilitate gene switching, expression, expansion and generations of new variants [53], [55]. Because we found an enrichment of TcS pseudogenes within subtelomeres (Figure 4C), we speculate that these *T. cruzi* regions have also been subjected to intense rearrangement. *T. cruzi* does not undergo antigenic variation but instead co-expresses several variable surface proteins, among which is TcS [59]. Nevertheless, the subtelomeric location of TcSgroupII may facilitate the generation of new variants. In fact, *in silico* simulations suggested that both mutation and gene conversion may contribute to the generation of diversity in the TcS family [60], [61]. Gene conversion may be frequent in subtelomeric regions, and therefore could promote a faster diversification of TcSgroupII. This scenario may be particularly important for this group because several of its members have been implicated in host cell attachment/invasion, and *T. cruzi* has the ability to infect a broad range of host cells. Therefore, it is possible that the large repertoire of peptides derived from TcSgroupII may contribute to this phenomenon.

Co-expression of several members of the TcS family has been described in the mammalian stages of the parasite [59]. Here, we show that the levels of expression are not homogeneous between and within the TcS groups (Figure 5). It is well known that the 3′UTRs are implicated in the control of the gene expression of several *T. cruzi* genes that are regulated during the life cycle. Although we have not mapped the 3′UTRs of the genes selected for expression analysis, for a few genes, it was possible to find a correlation between the expression profile and the sequence similarity in their 3′ flanking regions. For example, SAPA and TCNA genes, which are both active trans-sialidases expressed in trypomastigotes, have almost identical 3′ flanking regions (Figure 6). On the other hand, the 3′ flanking sequences of the genes TcS8 and TcS25 are quite similar (75% identity) despite the fact that their pattern of expression is very distinct (Figures 5 and 6). In this case, it is possible that cis-acting regulatory elements present in regions other than the 3′UTR may modulate their expression. It is also unclear what the proportion of the total TcS repertoire is expressed and whether the repertoire and/or the level of expressed genes may change during the parasite infection. High-throughput RNA sequencing approaches will clarify these questions.

We have also investigated the antigenic profile of peptides derived from distinct groups of the TcS family (Figure 7). Besides the known epitopes derived from the repetitive sequences of the SAPA and TcTS13 proteins, new B-cell epitopes were identified in members from both previously described and new TcS groups. Nine of the 14 reactive peptides are found in more than one TcS member. Specifically, the highly reactive peptide C3 (Figure 7) occurs in the largest number of proteins (60 in total) including members of the two new TcS groups V and VI. Also, similar but not identical sequences of this peptide were found in more than 150 TcS members. The cross-reaction among several epitopes and the sequence variability of the TcS family might contribute to the simultaneous presence of B-cell related epitopes during an infection. In fact, it has been proposed that cross-reactivity among the *T. cruzi* epitopes could be an evasion mechanism that drives the immune system into a series of spurious and non-neutralizing antibody responses [62]. In this regard, it has been shown that subtle differences at amino acid positions in or around the active site of the TcS proteins that have trans-sialidase activity might delay the immune response and avoid inhibiting the complete enzymatic makeup of the parasite [63]. This scenario may represent an evolutionary pressure driving the diversification of TcSgroupI, which harbors the active trans-sialidases. Whether a similar mechanism is involved in the diversification of the other TcS groups remains to be addressed.

The diversity of the TcS family may be even greater than reported here since the current assembly of the CL Brener genome is fragmented [2], [20], and therefore additional TcS genes may not be part of the dataset analyzed in this study. Nevertheless, based on the nearly complete repertoire of TcS sequences, we can now design probes and antibodies specific for each group, to be employed in more assertive strategies to investigate the role of this complex family during *T. cruzi* infection.

## Supporting Information

Figure S1 **Partial alignment of active trans-sialidase proteins.** FRIP and Asp-box motifs, and critical amino acids residues involved in trans-sialidase activity are shaded in gray. The amino acid positions are relative to the first methionine. Only N-terminal region of the active trans-sialidase proteins is shown.
(DOCX)

Figure S2 **Multidimensional scaling plot of the TcS proteins indicating the presence of characteristic TcS motifs.** TcS proteins with the motifs are represented by red dots. (A) SXDXGXTW motif; (B) VTVXNVXLYNR motif; (C) SXDXGXTW motif allowing 1 mismatch; (D) sequences with VTVXNVXLYNR motif found in the alignment block of the 505 TcS derived from the eight clusters identified in this study; (E) FRIP (XRXP) motif. X represents any amino acid.
(DOCX)

Figure S3 **Prototype of each TcS protein.** The peptide signal is represented in gray, FRIP in green, Asp-box in blue, VTVXNVXLYNR in red, repeats in black and GPI anchor addition site in orange.
(TIF)

Figure S4 **Divergent CRP – complement regulatory proteins.** Protein sequences involved in the regulation of complement system identified by Beucher and Norris (2008). Sequences were mapped on the MDS showed in Figure 1. HSG sequences (high similarity group) and LSG sequences (low-similarity group) are indicated by red and black squares, respectively.
(DOCX)

Table S1 **Primers used in the Real-time RT-PCR reactions.**
(DOC)

Table S2 **TcS peptides analyzed by immunoblotting.**
(DOCX)

Table S3 **List of members of each TcS group.**
(XLS)

Table S4 **List of TcS repeats.**
(XLSX)
