# Supplementary material for: Genomic Analyses, Gene Expression and Antigenic Profile of the Trans-Sialidase Superfamily of Trypanosoma cruzi Reveal an Undetected Level of Complexity
Source: PLoS One. 2011 Oct 19;6(10):e25914. doi: 10.1371/journal.pone.0025914 (PMC3198458; doi:10.1371/journal.pone.0025914)
Supplement: Figure S1 — Partial alignment of active trans-sialidase proteins. FRIP and Asp-box motifs, and critical amino acids residues involved in trans-sialidase activity are shaded in gray. The amino acid positions are relative to the first methionine. Only N-terminal region of the active trans-sialidase proteins is shown. (DOCX) [file pone.0025914.s001.docx]

**Figure S1. Partial alignment of active trans-sialidase proteins.**
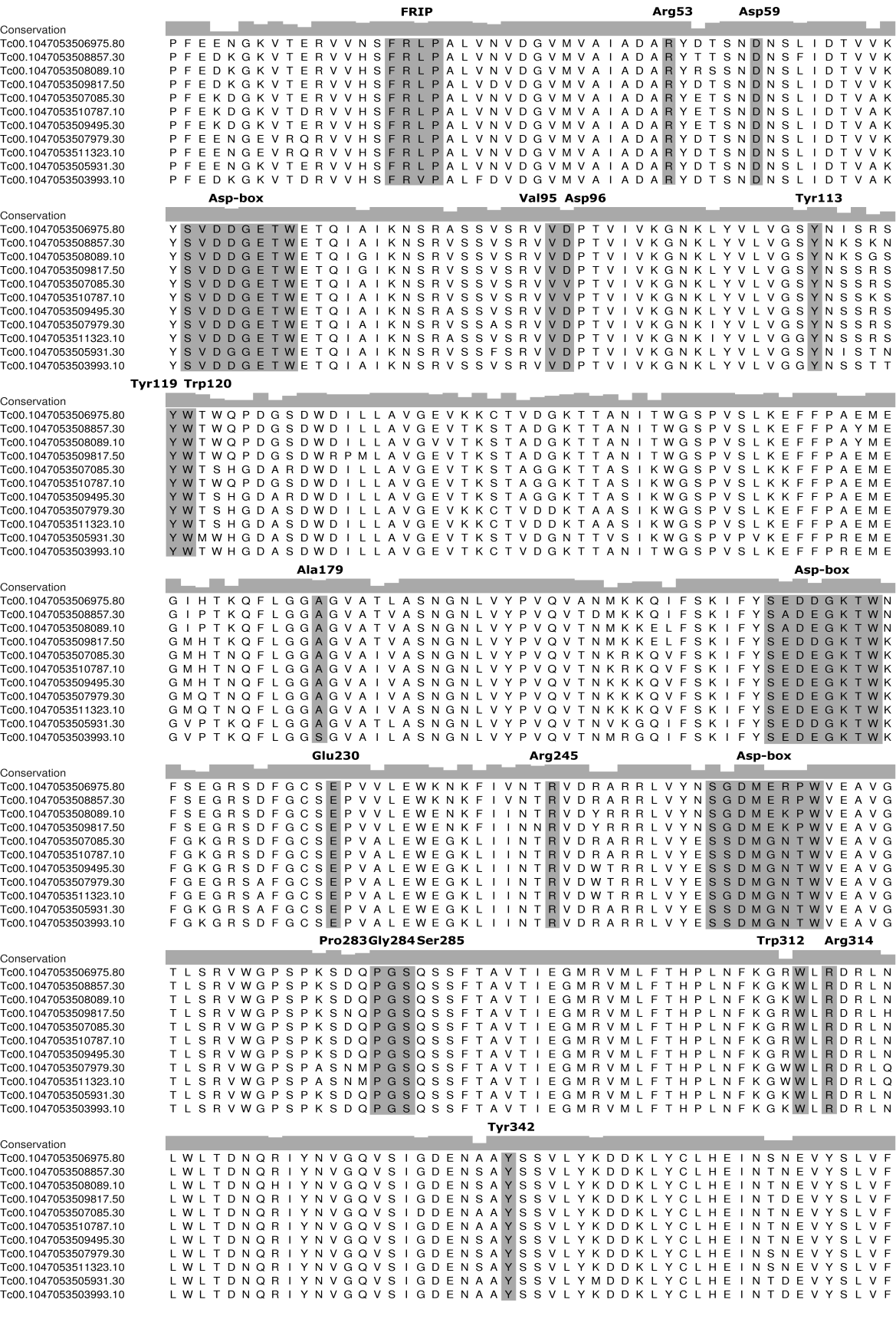


**Figure S1: Partial alignment of active trans-sialidase proteins.** FRIP and Asp-box motifs, and critical amino acids residues involved in trans-sialidase activity are shaded in gray. The amino acid positions are relative to the first methionine. Only N-terminal region of the active trans-sialidase proteins is shown.
