# Supplementary material for: Genomic Analyses, Gene Expression and Antigenic Profile of the Trans-Sialidase Superfamily of Trypanosoma cruzi Reveal an Undetected Level of Complexity
Source: PLoS One. 2011 Oct 19;6(10):e25914. doi: 10.1371/journal.pone.0025914 (PMC3198458; doi:10.1371/journal.pone.0025914)
Supplement: Figure S2 — Multidimensional scaling plot of the TcS proteins indicating the presence of characteristic TcS motifs. TcS proteins with the motifs are represented by red dots. (A) SXDXGXTW motif; (B) VTVXNVXLYNR motif; (C) SXDXGXTW motif allowing 1 mismatch; (D) sequences with VTVXNVXLYNR motif found in the alignment block of the 505 TcS derived from the eight clusters identified in this study; (E) FRIP (XRXP) motif. X represents any amino acid. (DOCX) [file pone.0025914.s002.docx]

**
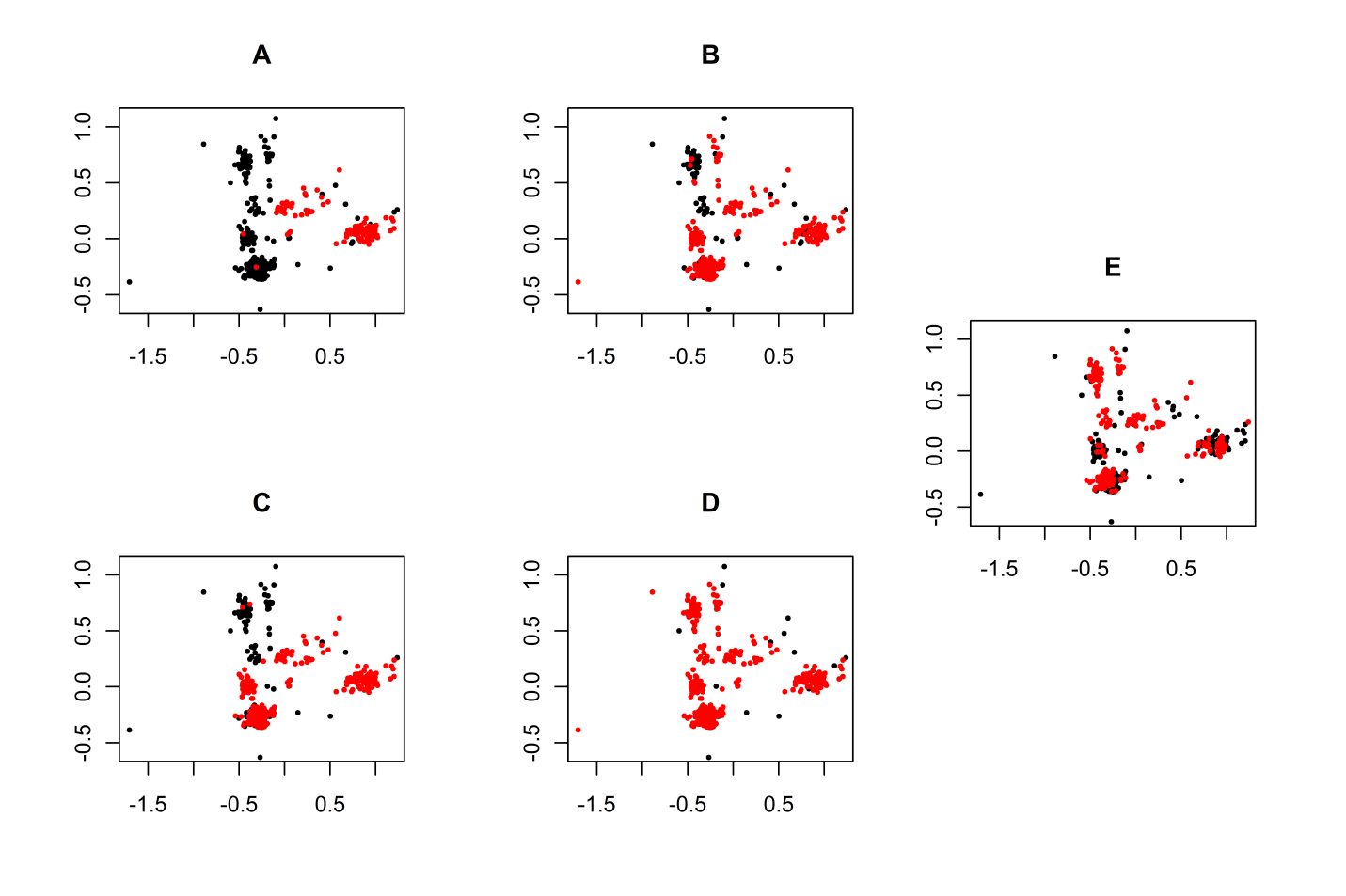
** **Figure S2. Multidimensional scaling plot of the TcS proteins indicating the presence of characteristic TcS motifs.** TcS proteins with the motifs are represented by red dots. (A) SXDXGXTW motif; (B) VTVXNVXLYNR motif; (C) SXDXGXTW motif allowing 1 mismatch; (D) sequences with VTVXNVXLYNR motif found in the alignment block of the 505 TcS derived from the eight clusters identified in this study; (E) FRIP (XRXP) motif. X represents any amino acid.
