# Supplementary material for: Genomic Analyses, Gene Expression and Antigenic Profile of the Trans-Sialidase Superfamily of Trypanosoma cruzi Reveal an Undetected Level of Complexity
Source: PLoS One. 2011 Oct 19;6(10):e25914. doi: 10.1371/journal.pone.0025914 (PMC3198458; doi:10.1371/journal.pone.0025914)
Supplement: Figure S4 — Divergent CRP – complement regulatory proteins. Protein sequences involved in the regulation of complement system identified by Beucher and Norris (2008). Sequences were mapped on the MDS showed in Figure 1. HSG sequences (high similarity group) and LSG sequences (low-similarity group) are indicated by red and black squares, respectively. (DOCX) [file pone.0025914.s004.docx]

**Figure S4. Divergent CRP – complement regulatory proteins.** Protein sequences involved in the regulation of complement system identified by Beucher and Norris (2008). Sequences were mapped on the MDS showed in Figure 1. HSG sequences (high similarity group) and LSG sequences (low-similarity group) are indicated by red and black squares, respectively.
