# Supplementary material for: Genomic Analyses, Gene Expression and Antigenic Profile of the Trans-Sialidase Superfamily of Trypanosoma cruzi Reveal an Undetected Level of Complexity
Source: PLoS One. 2011 Oct 19;6(10):e25914. doi: 10.1371/journal.pone.0025914 (PMC3198458; doi:10.1371/journal.pone.0025914)
Supplement: Table S1 — Primers used in the Real-time RT-PCR reactions. (DOC) [file pone.0025914.s005.doc]

**Table S1.** Primers used in the Real-time RT-PCR reactions.

| Locus_id | TcS group | Gene code | Primer Forward (5’ 🡪 3’) | Primer Reverse (5’ 🡪 3’) |
| --- | --- | --- | --- | --- |
| Tc00.1047053506471.120 | TcSgroupII dark green | TcS5 | CGGCGTCACTTCTATTCT | CGTCTTATTCGGCACAAG |
| Tc00.1047053506129.30 | TcSgroupII dark green | TcS27 | AGGCTCCGAGTAATAACAC | CATCGTCATCCACCTTCTA |
| Tc00.1047053507047.40 | TcSgroupII dark green | TcS29 | AGTCCTGAATACCTCTTCTG | AGTAATCGTCATTATCATCATCAA |
| Tc00.1047053511911.60 | TcSgroupIII light blue | TcS8 | GGAGAAGTTGGAGGACAT | CCATCATTACAGTCACTAAGC |
| Tc00.1047053507723.50 | TcSgroupIV magenta | TcS9 | GGTCTCGGAATCAGAAGA | CTTGAAGATGTGGCAGATG |
| Tc00.1047053510307.284 | TcSgroupIV magenta | TcS33 | CAGACCTCATTATGCCAATAG | GCTCATAGAGACGGAACT |
| Tc00.1047053510013.50 | TcSgroupIV magenta | TcS34 | CATTGGACCGTTATTTGCT | TTCATCCAGTTGCTCAGT |
| Tc00.1047053508221.750 | TcSgroupV red | TcS15 | CTTCCGTTACTACTCCTTCA | CTCCTCTTGTCTCCTCATC |
| Tc00.1047053508455.20 | TcSgroupV red | TcS21 | GATGATAAGGAACAAGTGGAAT | GACTTCACCGTTCTTCTTC |
| Tc00.1047053509377.20 | TcSgroupVII orange | TcS32 | CTATGTGCTGCCTATTCAAG | CATCCGTAATGACTCGTATG |
| Tc00.1047053510847.10 | TcSgroupVIII purple | TcS24 | CACGCTGAATGAAGACAATA | TTCCTCCTCCTCTGGTAT |
| Tc00.1047053503907.10 | TcSgroupVIII purple | TcS25 | TACAACCGCACAATCAGTA | GTCTCCGCTTCCTCAATA |
