# Supplementary material for: Genomic Analyses, Gene Expression and Antigenic Profile of the Trans-Sialidase Superfamily of Trypanosoma cruzi Reveal an Undetected Level of Complexity
Source: PLoS One. 2011 Oct 19;6(10):e25914. doi: 10.1371/journal.pone.0025914 (PMC3198458; doi:10.1371/journal.pone.0025914)
Supplement: Table S2 — TcS peptides analyzed by immunoblotting. (DOCX) [file pone.0025914.s006.docx]

**Table S2.** TcS peptides analyzed by immunoblotting

| **Peptide position in the membrane** | **TcS group** | **Peptide sequence** | **Number of TcS proteins containing the peptide sequence*** |
| --- | --- | --- | --- |
| A1 | V | KGTESSPQRIDTQSD | 1 |
| A2 | VIII | HNEASEGYEDDEYDE | 1 |
| A3 | VIII | DEEEETTQEVEEAPR | 2 |
| A4 | VIII | NEEETETDGSSGSAS | 1 |
| A5 | VIII | NQSRTKPPVEDTNER | 1 |
| A6 | VIII | ELNEYDPDEEEEEIV | 1 |
| A7 | VIII | VSEPAIATESAGASR | 1 |
| A8 | VIII | MQRGSDLHPQDPQPA | 1 |
| A9 | VII | ATLSSRSQHSPAQTS | 1 |
| A10 | VII | GSSERRKEGRRESGH | 1 |
| B1 | VII | GGGDGEEKEFGWDQG | 1 |
| B2 | VII | CEAASADGSSGEGKA | 1 |
| B3 | VII | LSPAPEAASGHKSPD | 2 |
| B4 | VII | QRKDAQDRSSEEENK | 2 |
| B5 | IV | AEPKPAEPKPAEPKP | 3 |
| B6 | IV | ASGGAPSTPAVSESE | 3 |
| B7 | IV | SAEPKPAEPKSAEPK | 5 |
| B8 | IV | VTIPPPERKSAKAAA | 3 |
| B9 | IV | ESRPEEPEPAREGTA | 2 |
| B10 | IV | GGAPSTPAESRPAEP | 2 |
| C1 | IV | NGSSDPSDGAPSTPA | 2 |
| C2 | V, VI | TQQPSVGTPATADTN | 7 |
| C3 | II, IV, V, VI | RRVTGSSGRRREGGE | 60 |
| C4 | VI | NTPTTEGEGQDGPTV | 2 |
| C5 | VI | GTPHAGQEPLNGGEG | 3 |
| C6 | VI | GKPPDGNADVDVSPS | 2 |
| C7 | V, VI | DANTPTTEGEGQYGP | 3 |
| C8 | VI | AVGGSTSPSTPSTIT | 2 |
| C9 | III | EKDTTPSPQNQGLSP | 3 |
| C10 | III | EQTVDPEERKDTNPH | 5 |
| D1 | III | LAPTAGEGSPQKAPE | 2 |
| D2 | III | SEKDATPSPQKQDLS | 3 |
| D3 | III | TAAHEPSTDPETAQG | 3 |
| D4 | III | TKAAAAPEAEVSAPE | 2 |
| D5 | I | STPVDSSAHGTPSTP | 3 |
| D6 | I | AHGTPSTPVDSSAHG | 2 |
| D7 | I | DSSAHGTPSTPVDSS | 3 |
| D8 | I | GTPSTPVDSSAHGTP | 2 |
| D9 | I | HPFTLGTPPRDGVPA | 1 |
| D10 | I | ITAPTHPDEDTPSAS | 2 |

*Counts were performed in the dataset used in this study
